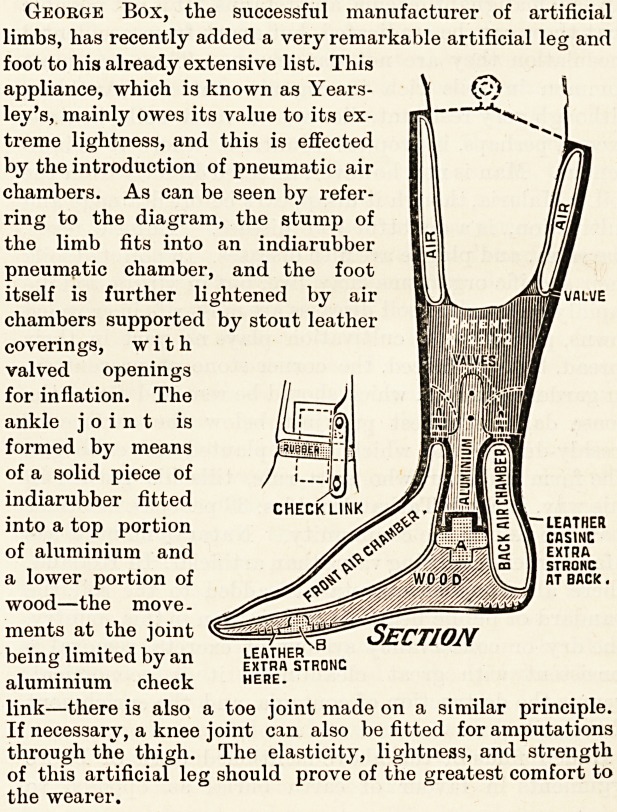# New Appliances and Things Medical

**Published:** 1899-08-05

**Authors:** 


					NEW APPLIANCES AND THINGS MEDICAL.
[We shall be glad to receive, at our Office, 28 & 29, Southampton Street, Strand, London, W.O., from the manufacturers, specimens of all new
preparations and appliances which may be brought out from time to time.]
ASPIRIN?A NEW SUBSTITUTE FOR SALICYLIC
ACID AND SALICYLATE OF SODIUM.
(The Bayer Co., Limited, 19, St. Dunstan's Hill,
London, E.C.)
Aspirin is the acetic-ester of salicylic acicl, and is, we
believe, the latest of the acetic-ester substitution compounds
which we owe to the above famous German chemists. It is a
white crystalline powder, sparingly soluble in water, readily
so in alcohol. It is insoluble in the stomach, and only forms
a solution in the alkaline juices of the intestine. Con-
sequently it lacks the irritating properties of salicylic acid,
and the salicylates on the walls of the stomach, and, further,
it has no unpleasant taste. It is claimed for it that no
singing in the ears follows its use, owing to its slow solubility.
This, which may be a virtue in many cases, may, however,
be a source of objection 'in very acute cases in which rapid
absorption is the main object of the physician. Aspirin, on
its chemical form, certainly deserves a thorough clinical
trial. The reported observations up to the present seem
highly satisfactory. The dose is about 15 grains.
PIPERAZINE CITRATE AND UROTROPINE
YAR ALETTES.
(Alfred Bisiiop, Limited, Mile End New Town,
London.)
Piperazine as an eliminant of uric acid from the system
deservedly bears a high reputation. There are, however,
objections to the use of the base as it is hydroscopic and
volatile, and hence not constant in strength. The citrate of
piperazine appears to be free from these objections, and
therefore Messrs. Bishop, Limited, have wisely selected this
salt for incorporation in their effervescent varalettes. In
each varalette there are three grains of the citrate, which is
equivalent to two and a half grains of the base piperazine.
By using the varalettes a neutral salt may be obtained in
solution ; the dose is accurate and constant, and easily
administered at all times of the day, as a small bottle of
varalettes can be easily carried in the pocket. Urotropine,
which is admittedly a very valuable solvent of uric acid, is
also supplied in the form of effervescent varalettes, four
grains being contained in each. Urotropine is a combination
of formalin and ammonia, and as such is a powerful anti-
septic. It is apparently non-toxic in doses up to 25 grains,
and non-irritant to the stomach. It is very soluble in water
and should always be administered in dilute solution, and
in this respect the value of the varalette is particularly to be
commended.
NEW ARTIFICIAL PNEUMATIC LEG.
(George Box, 144, Oxford Street, Manchester.)
George Box, the successful manufacturer of artificial
limbs, has recently added a very remarkable artificial leg and
foot to his already extensive list. This
appliance, which is known as Years-
ley's, mainly owes its value to its ex-
treme lightness, and this is effected
by the introduction of pneumatic air
chambers. As can be seen by refer-
ring to the diagram, the stump of
the limb fits into an indiarubber
pneumatic chamber, and the foot
itself is further lightened by air
chambers supported by stout leather
coverings, with
valved openings
for inflation. The
ankle joint is
formed by means
of a solid piece of
indiarubber fitted
into a top portion
of aluminium and
a lower portion of
wood?the move-
ments at the joint
being limited by an
aluminium check
link?there is also a toe joint made on a similar principle.
If necessary, a knee joint can also be fitted for amputations
through the thigh. The elasticity, lightness, and strength
of this artificial leg should prove of the greatest comfort to
the wearer.
George Box, the successful manufacturer of artificial
limbs, has recently added a very remarkable artificial leg and
foot to his already extensive list. This
appliance, which is known as Years-
ley's, mainly owes its value to its ex-
treme lightness, and this is effected
by the introduction of pneumatic air
chambers. As can be seen by refer-
ring to the diagram, the stump of
the limb fits into an indiarubber
pneumatic chamber, and the foot
itself is further lightened by air
chambers supported by stout leather
coverings, with
valved openings
for inflation. The
ankle joint is
formed by means
of a solid piece of
indiarubber fitted
into a top portion
of aluminium and
a lower portion of
wood?the move-
ments at the joint
being limited by an ^^stfTonc
aluminium check here.
link?there is also a toe joint made on a similar principle.
If necessary, a knee joint can also befitted for amputations
through the thigh. The elasticity, lightness, and strength
of this artificial leg should prove of the greatest comfort to
the wearer.

				

## Figures and Tables

**Figure f1:**